# A Natural Mutation Involving both Pathogenicity and Perithecium Formation in the *Fusarium graminearum* Species Complex

**DOI:** 10.1534/g3.116.033951

**Published:** 2016-09-27

**Authors:** Haruhisha Suga, Koji Kageyama, Masafumi Shimizu, Misturo Hyakumachi

**Affiliations:** * Life Science Research Center, Gifu University, 501-1193, Japan; † River Basin Research Center, Gifu University, 501-1193, Japan; ‡ Faculty of Applied Biological Sciences, Gifu University, 501-1193, Japan

**Keywords:** *Gibberella zeae*, ascomycetous fungus, pathogenicity, ascocarp, gene mapping

## Abstract

Members of the *Fusarium graminearum* species complex (*Fg* complex or FGSC) are the primary pathogens causing *Fusarium* head blight in wheat and barley worldwide. A natural pathogenicity mutant (strain 0225022) was found in a sample of the *Fg* complex collected in Japan. The mutant strain did not induce symptoms in wheat spikes beyond the point of inoculation, and did not form perithecia. No segregation of phenotypic deficiencies occurred in the progenies of a cross between the mutant and a fully pathogenic wild-type strain, which suggested that a single genetic locus controlled both traits. The locus was mapped to chromosome 2 by using sequence-tagged markers; and a deletion of ∼3 kb was detected in the mapped region of the mutant strain. The wild-type strain contains the FGSG_02810 gene, encoding a putative glycosylphosphatidylinositol anchor protein, in this region. The contribution of FGSG_02810 to pathogenicity and perithecium formation was confirmed by complementation in the mutant strain using gene transfer, and by gene disruption in the wild-type strain.

*Fusarium* head blight (FHB), or scab, is one of the most destructive and economically important diseases of wheat and barley worldwide. The major etiological agent, *Fusarium graminearum* Schwabe (*F. graiminarum* Schwabe), is a haploid ascomycetous fungus. This pathogen causes quantitative losses in yield, and diseased grains may be contaminated with significant levels of mycotoxins such as deoxynivalenol (DON) and zearalenone, which are harmful to humans and animals (Desjardins *et al.* 1993; Windels 2000). *F. graiminarum* can reproduce sexually by self-crossing, and also by outcrossing. Ascospores from sexual reproduction and conidia from asexual reproduction are believed to be the main *F. graminearum* inoculums infecting flowering wheat heads (Francl *et al.* 1999).

Molecular phylogenetic analyses using worldwide collections of *F. graminearum* revealed that the *F. graminearum* species complex (*Fg* complex) includes at least 16 biogeographically structured species (O’Donnell *et al.* 2000, 2004, 2008; Starkey *et al.* 2007; Yli-Mattila *et al.* 2009; Sarver *et al.* 2011); Of these, only six species, and three species groups, are morphologically recognizable (Aoki *et al.* 2012). Reciprocal monophyly of the *Fg* complex species indicates that interspecific hybridization in nature is relatively limited; however, crossing between strains of different species has been attained in the laboratory (Bowden and Leslie 1999). Previously, a genetic map was constructed by interspecific crossing *F. graminearum* s. str. with *F. asiaticum*, and segregation distortion was observed on this crossing (Jurgenson *et al.* 2002). A genetic map with sequence-tagged markers was also generated by intraspecific crossing *F. graminearum* s. str. with *F. graminearum* s. str. to complement the whole genome sequence of *F. graminearum* s. str, resulting in the prediction of 11,640 genes (Gale *et al.* 2005; Cuomo *et al.* 2007).

Various forward and reverse genetic approaches have been applied to identify genes that contribute to *Fg* complex pathogenicity. Urban and Hammond-Kosack (2013) have summarized 159 known pathogenicity genes. These genes have been deduced by functional annotation to be involved mainly in (1) metabolism, (2) cellular communication/signal transduction, (3) cell rescue, defense, and pathogenicity/adaptation to nutrient conditions, and (4) transcription (Urban and Hammond-Kosack 2013). Trichothecenes, such as DON, are known to inhibit eukaryotic protein synthesis (Pestka and Smolinski 2005). A non-DON-producing mutant, in which the *tri5* gene is disrupted, can cause an initial infection on wheat spikes, but the infection does not extend beyond this site (Bai *et al.* 2002). At the molecular level, however, no single gene can fully account for the *Fg* complex’s wheat head infection and symptom development, and thus observation of aberrant phenotypic behavior for each gene disruptant would be required for comprehensive understanding of infection by the *Fg* complex.

Urban and Hammond-Kosack (2013) split the phenotypic process of infection (as observed for the wild-type *Fg* complex) into seven stages (A–G). Fungal invasion into the adjacent spikelet through the rachis node (from the initially infected wheat spikelet) is one of the critical stages for symptom development in the entire wheat head. The mutant that fails at stage E (entry into the rachis node as indicated by the internal rachis) stands out because visible signs of disease do not spread beyond the inoculated wheat spikelet when artificial point inoculation is performed. This type of mutant has been recognized in artificially created mutants, and sometimes also in natural *Fg* complex isolates. Associations between pathogenicity to wheat and phylogenetic lineages, trichothecene chemotype, and genetic diversity, have been investigated using *Fg* complex isolates (O’Donnell *et al.* 2000; Carter *et al.* 2002; Logrieco *et al.* 1990). Pathogenic variation among isolates has been reported, and a stage E mutant, one of several possible strains, was prominent (O’Donnell *et al.* 2000).

Cumagun *et al.* (2004) used a natural nonpathogenic isolate of *F. asiaticum* to map the genomic region associated with pathogenicity. Cumagun *et al.* (2004), using quantitative trait locus (QTL) analysis, mapped one locus for pathogenicity, and two loci for aggressiveness on a previously constructed linkage group; however, the genes and mutations for these traits remain unknown. We also found several stage E mutants in our Japanese *Fg* complex sample (Suga *et al.* 2008). Disease symptoms were induced at the inoculated wheat spike and its rachis node, but the infection did not spread when their conidia were injected. In addition, these mutants also affected perithecium formation on carrot agar medium. In this study, we define these phenotypic mutations as nonpathogenic and nonperithecium-forming.

To comprehensively understand infection by the *Fg* complex, the objectives of this study were (1) to map the genomic region associated with nonpathogenic and nonperithecium-forming mutations, (2) to identify the gene that confers this phenotype by using the whole genome sequence of *F. graminearum* s. str. (Cuomo *et al.* 2007), and (3) to reveal what type of mutation occurs naturally in the *Fg* complex. As far as we know, this is the first report to clarify a natural mutation involving both pathogenicity and perithecium formation in the *Fg* complex.

## Materials and Methods

### Strains and culture conditions

Regarding the *Fg* complex in Japan, the wild-type strain *F. graminearum* s. str. 0407011 (Fg0407011), showing pathogenicity and perithecium formation, and the mutant-type strain *F. asiaticum* 0225022 (Fa0225022), showing nonpathogenicity and nonperithecium formation, were used (Suga *et al.* 2008). Although they belong to different species, the Fg0407011 strain was chosen as a crossing partner of the Fa0225022 strain because crossing between *F. graminearum* s. str. and *F. asiaticum* has been confirmed (Jurgenson *et al.* 2002), thereby allowing molecular markers to be established on the basis of nucleotide polymorphisms.

Conidia used for the pathogenicity test were collected from 10-d-old cultures on synthetic nutrient agar (SNA) under black light, and suspended in sterile distilled water containing 1% (v/v) Triton X-100. *F. asiaticum* strain 0444002 (Fa0444002) (Suga *et al.* 2011) was used to obtain a wild-type sequence of the target region of *F. asiaticum*. The strains and transformants were maintained on potato dextrose agar (PDA) and kept at –80° in 50% glycerol for long-term storage.

### Crossing

Members of the *Fg* complex reproduce sexually. Both self-crossing and outcrossing are capable in the *Fg* complex (Bowden and Leslie 1999). Outcrossing with nitrate-nonutilizing (nit) mutants of the parental strain was conducted according to a mycelial plug crossing method (Bowden and Leslie 1999). The progenies derived from outcrossing were needed for mapping, and were phenotypically recognizable when different type of nit mutants were paired. Nit mutants were generated from the parental strains on PDA containing 1.5–5.0% KClO_3_, and a *nit1*, *nit3*, or Nit M phenotype was determined by growth on different nitrogen sources (Correll *et al.* 1987). Plugs of a Nit M mutant of Fg0407011, and a *nit1* mutant of Fa0225022, were placed on opposite sides of a 90-mm Petri dish containing carrot agar (Klittich and Leslie 1988).

After incubation at 25° for 1 wk under black light, 1.5 ml of 2.5% (v/v) Tween 60 solution was added, and the aerial mycelium was knocked down with a glass rod. Thereafter, the Petri dish was kept under the same conditions to form perithecia. After 2 wk, the Petri dish was reversed, and coupled to another Petri dish of minimal medium containing 0.05% (v/v) tergitol type NP-10, and 2% (w/v) l-sorbose instead of 3% sucrose (MMTS) (Bowden and Leslie 1999) to obtain colonies derived from discharged ascospores. Wild-type colonies, characterized by dense and aerial mycelium, and nit mutant-type colonies, with thin and little, or no, aerial mycelium, appeared on MMTS, and an individual wild-type colony was used as progeny in this study. Conidia produced on SNA under black light were spread on MMTS to obtain pure cultures of each progeny by single colony isolation.

### Pathogenicity test

The pathogenicity test was performed using the rapid-maturing dwarf wheat cultivar Apogee, which has high susceptibility to FHB (Mackintosh *et al.* 2006). Each plant, cultivated in a small pot until one head remained, was inoculated just after heading out with ∼1 × 10^2^ conidia added to the wounded lower spikelet. After inoculation, the plants were placed in a humidity box for 24 hr, and then transferred to a growth chamber (KG-50HLA; Koito Electric Industries, Shizuoka, Japan) maintained at 27°.

**Figure 1:**
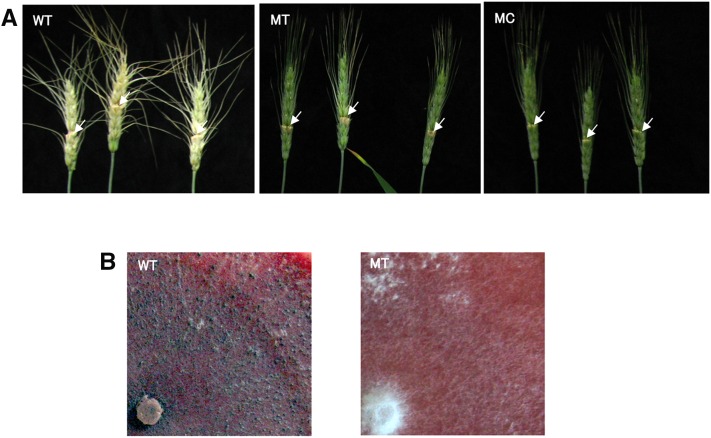
Disease and perithecium formation of the *Fusarium graminearum* species complex. (A) Pathogenicity test performed on wheat head with conidia of wild-type strain Fg0407011 (WT), mutant-type strain Fa0225022 (MT), and mock conidia (MC). Arrow indicates inoculated spikelet. (B) Perithecium formation assay on carrot agar plates for Fg0407011 (WT) and mutant-type strain Fa0225022 (MT).

Inoculation treatments consisted of one head for a progeny, and three heads for a transformant. Experimental treatments were repeated at least once for progenies and twice for transformants. Bleaching and/or necrosis of the spikelet 9 d after inoculation was observed. Pathogenicity was scored as pathogenic or nonpathogenic based on whether the symptoms were confined in the initially inoculated spikelet, as for the mutant Fa0225022, or whether they spread to the neighboring florets, as for the wild type, Fg0407011 (Figure 1). The pathogenicity of transformants was evaluated by the number of florets showing symptoms.

### Perithecium formation

Perithecium formation in self-crossing was assayed on carrot agar plates (Klittich and Leslie 1988) as described above, except that the mycelial plug of a single progeny was placed in the center of the 90-mm plate. Perithecium formation was observed until 3 wk after aerial mycelium knockdown (Figure 1).

### DNA extraction and PCR

Genomic DNA for PCR was extracted from 3- to 4-d-old mycelium (2–3 cm diameter) cultured on potato dextrose broth (PDB), as previously described (Suga *et al.* 2008). The final DNA pellet was dissolved in 200 μl of water, and 5 ng/μl yields of genomic DNA were estimated by comparison to DNA of known concentrations by agarose gel electrophoresis. Genomic DNA for Southern blot hybridization was extracted in a similar way with some modifications: >0.1 g of dry weight mycelium was ground in a mortar with a small amount of sea sand, and 5 ml of potassium ethyl xanthogenate (PEX) solution was added. RNase A treatment and phenol-chloroform extraction were performed after dissolving the DNA pellet in 200 μl of water.

### Mapping

Thirty-two variable number tandem repeat sequence (VNTR) loci for rough mapping were selected from 54 VNTR loci in the whole genome sequence of *F. graminearum* s. str. PH1 strain based on the results of length polymorphisms of PCR products between the parental strains (Suga *et al.* 2004). The PCR comprised 0.5 units of AmpliTaq DNA polymerase (Life Technologies, Carlsbad, CA), in a 20-μl reaction mixture containing 1× reaction buffer, 200 μM dNTPs, each primer at 1 μM, and 5 ng of genomic DNA. PCR was performed in an iCycler thermal cycler (Bio-Rad Laboratories, Hercules, CA) using the following cycling parameters: 94° for 2 min, 25 cycles of 94° for 1 min, 58° for 1 min, and 72° for 1 min. PCR products were analyzed and compared by electrophoresis on 2% MetaPhor agarose gel (Cambrex Bio-Science, Rockland, ME).

Data obtained with VNTR and the PCR-restriction fragment length polymorphism (RFLP) marker HS296/*Cla*I (described later in fine mapping) were processed using JoinMap v3.0 linkage map software (Van Ooijen and Voorrips 2001) to detect a significant association between pathogenicity and perithecium formation; the markers were processed using logarithm of odds (LOD) scores.

PCR-RFLP markers were developed for fine mapping at the protein coding gene, including introns between VNHK1049 and VNHK995 in the whole genome sequence of *F. graminearum* s. str. (Cuomo *et al.* 2007). Primers were designed with the primer designing tool in the Saccharomyces Genome Database (http://www.yeastgenome.org/), and restriction enzymes that showed polymorphism between parental strains were selected from *Hin*fI, *Hha*I, *Mse*I, *Hae*III, *Hinc*II, *Alu*I, *Sau*3AI, and *Rsa*I based on the results of 1.5% agarose gel electrophoresis.

The PCR conditions were the same for VNTR markers except that the cycling parameters were 94° for 2 min, 25 cycles of 94° for 1 min, 63 or 68° for 2 min, and 72° for 1 min. A single PCR product was confirmed by electrophoresis on 1.0% agarose gel before restriction enzyme digestion. If there was no polymorphism with the tested restriction enzyme, PCR products were sequenced directly by BigDye terminator v3.1 cycle sequencing kits (Life Technologies), and then run on an ABI 3100 genetic analyzer (Life Technologies), as previously described by Suga *et al.* (2008). Restriction enzymes were selected by computational PCR-RFLP with Genetyx version 4.0 software (Genetyx, Tokyo, Japan) based on the nucleotide sequence of the parental strains.

### Detection of missing region

Similar to PCR-RFLP, PCR primers were designed upstream and downstream of the open reading frame of each gene in the genomic region between the HS369/*Hha*I and HS367/*Ava*I markers. A *Not*I recognition sequence was added to the 5′ end of the primer for cloning the PCR product into plasmid vector pCB1004 (Carroll *et al.* 1994). The PCR comprised 0.5 units of AccuTaq LA DNA Polymerase (Sigma, St. Louis, MO) in a 20-μl reaction mixture, containing 1× reaction buffer, 500 μM dNTPs, 2% dimethyl sulfoxide (DMSO), each primer at 0.4 μM (Supplemental Material, Table S1), and 5 ng of genomic DNA. PCR was performed in an iCycler thermal cycler (Bio-Rad Laboratories) using the following cycling parameters: 94° for 30 sec, 30 cycles of 94° for 15 sec, 51° for 20 sec, and 68° for 5 min.

The PCR product generated with the primer pair HS484/ HS451 was directly sequenced as described above by using primers HS484, HS512, HS513, HS514, HS515, HS516, and HS451 (Table S1). The sequences of Fa0225022 (HQ599308), Fg0407011 (HQ599310), and Fa0444002 (HQ599309) have been deposited in GenBank.

### Construction of the transformation vector

The FGSG_02809, FGSG_02810, and FGSG_02811 genes, including regions upstream and downstream of the open reading frame, were amplified from Fg0407011 by PCR using the HS458/HS459, HS450/HS451, and HS442/HS443 primer pairs, respectively (Table S1). PCR products treated with *Not*I were inserted into the *Not*I site in pCB1004 to create the respective transformation vectors pCB02809, pCB02810, and pCB02811 using DNA Ligation Kit Ver. 2 (Takara, Otsu, Japan).

The FGSG_02810 replacement vector pCR402810dis was constructed as follows. The PCR product from FGSG_02810 without *Not*I treatment was cloned into pCR4TOPO (Invitrogen, Carlsbad, CA) according to the manufacturer’s instructions to obtain plasmid pCR402810. The hygromycin-resistance cassette region (TrpC promoter and hygromycin phosphotransferase coding sequence) in pCB1003 (Sweigard *et al.* 1997) was amplified by PCR using primers HS708 and HS709 in which *Xho*I and *Sac*II recognition sequences were integrated, respectively (Table S1), using pCB1003 as the template DNA.

PCR was performed by using AmpliTaq DNA polymerase (Life Technologies), and the following cycling parameters: 94° for 2 min, 30 cycles of 94° for 1 min, 60° for 1 min, and 72° for 1 min. Amplicons treated with *Xho*I and *Sac*II were inserted into *Xho*I and *Sac*II sites in pCR402810 (pCR402810dis) using DNA Ligation Kit Ver. 2.

### Fungal transformation

Mung bean liquid media inoculated with tiny PDA blocks of fungal culture were reciprocally shaken for 2 d at 25° to produce budding cells. The cells were harvested by centrifugation after filtration through Kim Wipes (Kimberly-Clark, Tokyo, Japan), and then suspended in PDB at a final concentration of 5.0 × 10^5^/ml. The suspension was incubated at 25° with gentle shaking until the length of the germ tubes reached 2–3 times the size of the budding cells. The germlings were harvested by filtration and washed with 1.2 M NaCl. For protoplasting, germlings were suspended in an enzyme solution containing 20 mg/ml of lysing enzyme (Sigma), 10 mg/ml of Cellulase Onozuka RS (Yakult, Tokyo, Japan), 10 mg/ml of Zymolyase 20T (Seikagaku Kogyo, Tokyo, Japan), 10 mg/ml of β-Glucuronidase type H-1 (Sigma), and a small amount of chitinase (Sigma) in 1.2 M NaCl, and agitated gently at 30° for 4–6 hr.

After filtration through four layers of Kim Wipes, a fivefold volume of SE (1 M sorbitol, 50 mM EDTA, pH 8.0) was added, and then protoplasts were harvested by centrifugation at 700 × *g* for 10 min. Protoplasts were washed first in SE, and then in STC (1 M sorbitol, 25 mM Tris-HCl, pH 7.0, 25 mM CaCl_2_). The final protoplasts were suspended in STC at a concentration of ∼5.0 × 10^7^ per ml.

Transformation was performed according to the procedure of Wasmann and Van Etten (1996), with modifications as follows. Plasmid DNA was extracted by a Qiagen Midi Kit (Qiagen, Hilden, Germany) according to the manufacturer’s instructions. Transformation plates were incubated under light, and 5 ml of 1% water agar containing 250 μg of hygromycin B (Wako, Osaka, Japan) per milliliter was overlaid twice so that hygromycin-resistant colonies appeared on the first overlay. FGSG_02810 gene replacement mutants were screened by a lack of DNA amplification by PCR using the primer pair HS729/HS730 (Table S1) from the resulting transformants with pCR402810dis. The gene replacements in the mutants were further confirmed by Southern blot analyses.

### Reverse transcription (RT)-PCR

Total RNA was extracted from mycelium cultured in PDB for 4 d, as well as from a wheat head inoculated with the strain; ∼1 × 10^4^ conidia were inoculated to each wounded spikelet and placed in a humidity box for 48 hr. RNA extraction was performed with the RNeasy Plant Mini Kit (Qiagen) according to the manufacturer’s instructions, and then purified with Recombinant DNase I (Takara) and NucleoSpin RNA clean-up kit (Takara). RT-PCR was performed with a Titan One Tube RT-PCR Kit (Roche Diagnostics, Mannheim, Germany), and the primer pair HS601/HS602 (Table S1) according to the manufacturer’s instructions. PCR was performed in an iCycler thermal cycler (Bio-Rad) using the following cycling parameters: 50° for 30 min (and 94° for 2 min), 30 cycles of 94° for 1 min, 60° for 1 min, and 68° for 1 min. Genomic DNA instead of RNA was also used to compare RT-PCR products on 1.0% agarose gel electrophoresis.

### Southern blot hybridization

Southern blot hybridization was performed using a probe for a region in the FGSG_02810 gene that is missing in the Fa0225022 strain. A 186 bp-DNA fragment amplified from the Fg0407011 genome by PCR using primer pair HS601/HS602 was labeled with digoxigenin (DIG) by using a PCR DIG Probe Synthesis Kit (Roche Diagnostics). The probe was used after purification by Mini Quick Spin DNA Columns (Roche Diagnostics). Genomic DNA digested with *Cla*I (New England Biolabs, Beverly, MA) was subjected to agarose gel electrophoresis together with DNA Molecular Weight Marker III, DIG-labeled (Roche Diagnostics), and transferred to a nylon membrane. The signal was detected by using DIG High Prime DNA Labeling and Detection Starter Kit I, according to the manufacturer’s instructions.

### Data availability

The authors state that all data necessary for confirming the conclusions presented in the article are represented fully within the article.

## Results

### Rough mapping

In total, 100 progenies were obtained from crossing between the wild-type strain (Fg0407011) and the mutant-type strain (Fa0225022). Sixty-four and 36 progenies showed pathogenicity and perithecium formation (*i.e.*, wild-type), and nonpathogenicity and nonperithecium formation (*i.e.*, mutant-type), respectively. Nonpathogenic and nonperithecium formation were not segregated in the progenies, indicating that a single gene was responsible for the traits, although 1:1 segregation of the wild type and mutant type was rejected (*P* = 0.005, χ^2^ test).

**Figure 2:**
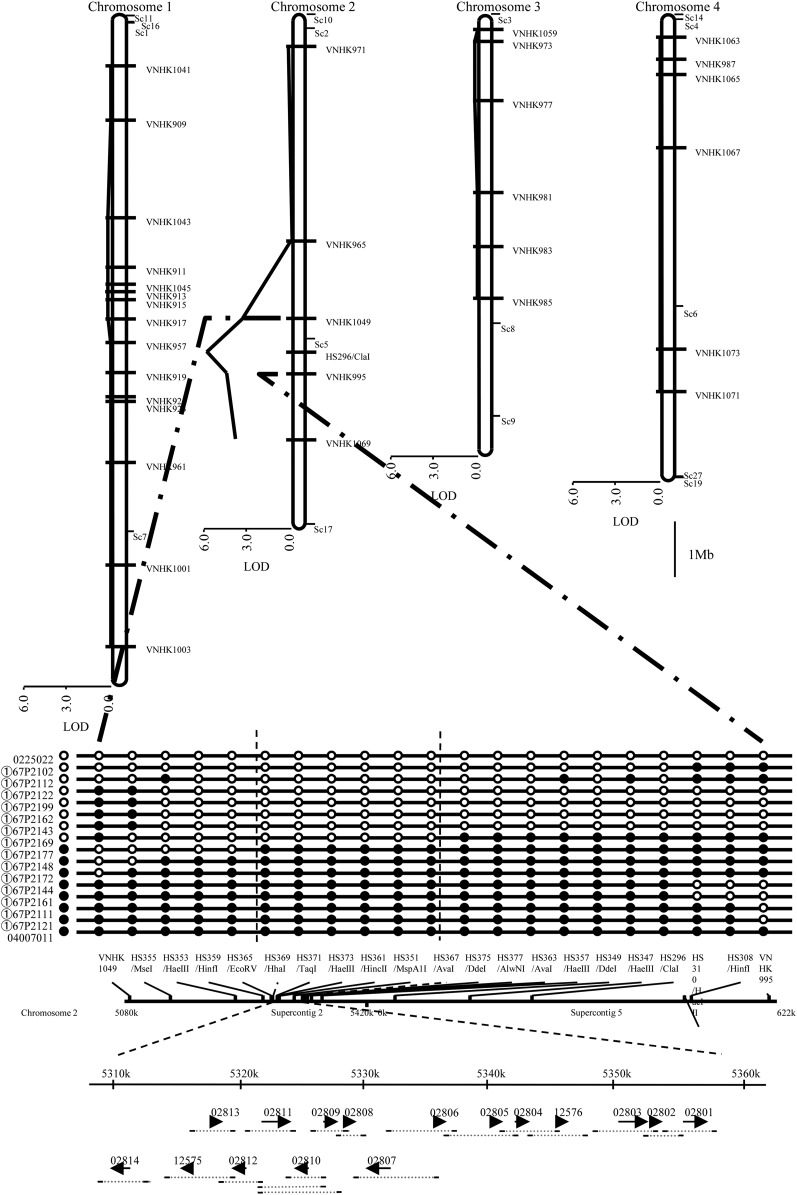
Mapping of nonpathogenicity and nonperithecium formation in the mutant strain Fa0225022. The chromosomal location of 32 VNTR markers, a PCR-RFLP marker (HS296/*Cla*I) in the MAT region, and supercontig (Sc) based on the whole genome sequence of *F. graminearum* s. str. PH1 is indicated at the top. The LOD value was obtained by analyzing 21 progenies from a crossing between the wild-type strain Fg0407011, and the mutant-type strain Fa0225022. The VNHK1049, VNHK995, VNHK1069, and HS296/*Cla*I markers on chromosome 2 showed high LOD values (>3.5). The result of fine mapping is indicated below. Fourteen progenies (①67P2102–①67P2121) that showed the Fg0407011-type allele at VNHK1049 and the Fa0225022 allele at VNHK995 (or vice versa) were used. White and black circles on lines indicate the Fa0225022 and Fg0407011-type allele, respectively; white and black circles next to the progeny’s name indicate mutant phenotype (nonpathogenic and nonperithecium formation) and wild-type phenotype (pathogenic and perithecium formation), respectively. The marker within the vertical dotted lines showed a perfect match between allele-type and phenotype in the 14 progenies. Genes within the region are shown as arrows above the FGSG gene ID number indicated. The dotted gray horizontal line below the arrows shows the PCR amplification test region for each gene, and the terminal bold lines represent the primers. The short, middle, and long dotted horizontal lines for FGSG_02810 indicate primer pairs HS450/HS451, HS484/HS451, and HS484/HS485, respectively.

Twenty-one progenies were analyzed with 32 VNTR markers. Three markers in chromosome 2 showed high LOD values (VNHK1049, 3.55; VNHK995, 4.57; and VNHK1069, 3.71) for the mutant phenotype (Figure 2). The mating type ideomorph (MAT) region essential for perithecium formation was found to be present at a distance of ∼360 kb to VNHK995 in the whole genome sequence of *F. graminearum* s. str., and the PCR-RFLP marker and HS296/*Cla*I in the MAT region had a higher LOD score (5.98) than that of VNHK995 (Figure 2).

### Fine mapping

All the remaining progenies were analyzed with VNHK1049, VNHK995, and HS296/*Cla*I markers. The HS296/*Cla*I marker showed the highest LOD among these, but the phenotype of one progeny differed from the results of marker type; progeny ①67P2169, with a mutant phenotype, had an Fg0407011-type allele at HS296/*Cla*I.

Fourteen progenies that had the Fg0407011-type allele at VNHK1049 and the Fa0225022-type allele at VNHK995, or vice versa, were used for further analysis. Five (HS355/*Mse*I, HS353/*Hae*III, HS351/MspA1I, HS349/*Dde*I, and HS347/*Hae*III) and two (HS308/*Hin*fI and HS310/*Hae*III) PCR-RFLP markers were newly developed for the genomic regions between VNHK1049 and HS296/*Cla*I, and between HS296/*Cla*I and VNHK995, respectively. Among these, the HS351/*Msp*A1I marker showed a perfect match between marker type and phenotype in the 14 progenies.

Therefore, PCR-RFLP markers (HS359/*Hin*fI and HS357/*Hae*III) were newly developed for the middle region between HS351/*Msp*A1I and adjacent PCR-RFLP markers (HS353/*Hae*III and HS349/*Dde*I). These markers did not show a perfect match between marker type and phenotype in the 14 progenies; therefore, new marker development was repeated in the same way until six serial markers that showed a perfect match between marker type and phenotype in the 14 progenies were found: HS369/*Hha*I, HS371/*Taq*I, HS373/*Hae*III, HS361/*Hin*cII, HS351/*Msp*A1I, and HS367/*Ava*I (Figure 2). A perfect match between marker type and phenotype in the 100 progenies was confirmed with HS369/*Hha*I and HS367/*Ava*I.

### PCR amplification of the target region

**Figure 3:**
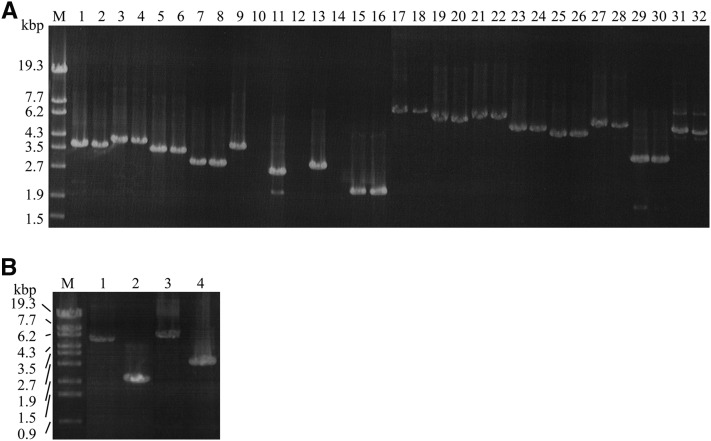
PCR amplification of the target region. (A) PCR product for 16 genes in the genomic region between the HS369/*Hha*I and HS367/*Ava*I markers. The amplification regions are shown in Figure 2. The expected size of PCR product for each gene is listed in Table S1. Size markers are shown in lane M. Each pair of lanes across lanes 1–32 are the results of Fg0407011 and Fa0225022, respectively, for FGSG_02814 (lanes 1 and 2), FGSG_12575 (lanes 3 and 4), FGSG_02813 (lanes 5 and 6), FGSG_02812 (lanes 7 and 8), FGSG_02811 (lanes 9 and 10), FGSG_02810 (lanes 11 and 12), FGSG_02809 (lanes 13 and 14), FGSG_02808 (lanes 15 and 16), FGSG_02807 (lanes 17 and 18), FGSG_02806 (lanes 19 and 20), FGSG_02805 (lanes 21 and 22), FGSG_02804 (lanes 23 and 24), FGSG_12576 (lanes 25 and 26), FGSG_02803 (lanes 27 and 28), FGSG_02802 (lanes 29 and 30), and FGSG_02801 (lanes 31 and 32). (B) PCR products obtained with the primer pairs HS484/HS451 and HS484/HS485. Shown are size markers (lane M), PCR product from Fg0407011 (lane 1) and Fa0225022 (lane 2) with HS484/HS451, and PCR product from Fg0407011 (lane 3) and Fa0225022 (lane 4) with HS484/HS485. Regions of amplification by HS484/HS451 and HS484/HS485 are indicated by the medium and long dotted horizontal lines below the FGSG_02810 gene in Figure 2. The primer pairs HS484/HS451 and HS484/HS485 amplify fragments of 5120 and 6042 bp DNA, respectively, based on the whole genome sequence of *F. graminearum* s. str. PH1.

PCR amplification was performed for the ORF and upstream and downstream regions of 16 genes present between the HS369/*Hha*I and HS367/*Ava*I markers based on annotation of the whole genome sequence of *F. graminearum* s. str. PH1 (Figure 2). The expected size of DNA was amplified for all 16 genes in Fg0407011, but the consecutive FGSG_02809, FGSG_02810, and FGSG_02811 genes were not amplified in Fa0225022 (Figure 3).

PCR amplification was performed with primers HS484 (forward) and HS485 (reverse) that had sequences complementary to the missing side primer of the FGSG_02812 and FGSG_02808 genes located next to the FGSG_02809 and FGSG_02811 genes, respectively, because successful PCR amplification was observed for these genes in Fa0225022 (Figure 3).

The expected size of DNA was amplified in Fg0407011, whereas a ∼3-kb smaller DNA fragment was amplified in Fa0225022 (Figure 3). The HS451 primer for FGSG_02810 was located nearer to the missing region in Fa0225022 as compared with the HS485 primer, and PCR amplification with the HS484 and HS451 primers in Fa0225022 also produced a ∼3-kb smaller DNA fragment than Fg0407011. By contrast, a size of DNA fragment similar to that in Fg0407011 was amplified from another *F. asiaticum* strain (Fa0444002).

**Figure 4:**
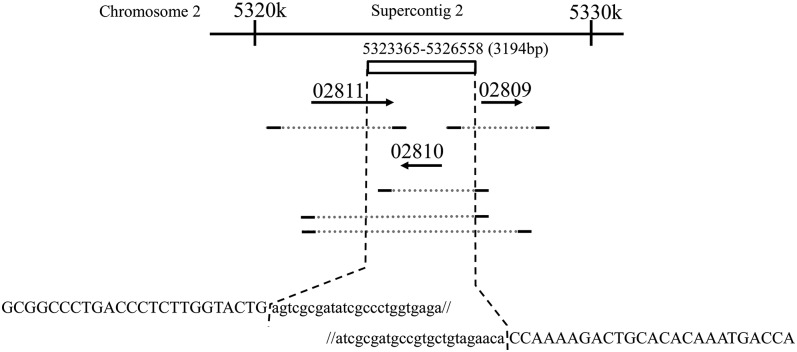
Schematic depiction of the missing genome region in Fa0225022. The genomic contexture is based on the whole genome sequence of *F. graminearum* s. str. PH1. The 3194-bp region (from 5323365 to 5326558 in Supercontig (2) in the genome of Fa0225022 is indicated by a white box; horizontal dotted lines show the borders of the missing region. Small letters indicate the *F. graminearum* s. str. PH1 sequence that is missing in Fa0225022. Genes are shown as arrows with the FGSG_ gene ID number indicated above. The dotted gray horizontal line below the arrows shows the region of the PCR amplification test in figure, and the terminal bold line represents the primer. The short, medium, and long dotted horizontal lines under FGSG_02810 indicate the region amplified with the primer pairs HS450/HS451, HS484/HS451, and HS484/HS485, respectively.

Sequencing of these DNA fragments, and the whole genome sequence of *F. graminearum* s. str. PH1 indicated that Fa0225022 has a deletion of 3194 bp between part of the FGSG_02811 gene and the upstream region of the FGSG_02809 gene, including the whole FGSG_02810 gene as compared with *F. graminearum* s. str. PH1 (Figure 4).

### Phenotype recovery by gene complementation

The deficiency of perithecium formation and pathogenicity in Fa0225022 was assumed to be due to malfunction of the FGSG_02809, FGSG_02810, and FGSG_02811 genes. Transformants of Fa0225022 were generated with a plasmid carrying the FGSG_02809, FGSG_02810, or FGSG_02811 gene of the wild-type strain Fg0407011 (pCB02809, pCB02810, or pCB02811, respectively). Both pathogenicity and perithecium formation were recovered in the transformants of the FGSG_02810 gene, but not in those of the FGSG_02809 and FGSG_02811 genes (Table 1).

**Table 1:** Pathogenicity test

Transformant	Pathogenicity Test*^a^*, Mean (± SD)		Perithecium Formation*^b^*
	1st Trial	2nd Trial	
Fa02811-1	1.0 (0.0)	1.0 (0.0)	—
Fa02811-2	1.0 (0.0)	1.0 (0.0)	—
Fa02811-3	1.0 (0.0)	1.0 (0.0)	—
Fa02810-1	10.0 (2.6)	13.0 (0.0)	+
Fa02810-2	4.3 (3.5)	5.0 (3.5)	+
Fa02810-4	5.3 (3.8)	2.0 (1.7)	+
Fa02809-1	1.0 (0.0)	1.3 (0.6)	—
Fa02809-2	1.0 (0.0)	1.0 (0.0)	—
Fa02809-3	1.0 (0.0)	1.0 (0.0)	—
Fg02810dis-5 (disruptant)	1.0 (0.0)	1.0 (0.0)	—
Fa02810dis-8 (disruptant)	1.0 (0.0)	1.0 (0.0)	—
Fg02810dis-36 (disruptant)	1.0 (0.0)	1.0 (0.0)	—
Fg02810dis-1 (ectopic)	13.0 (0.0)	13.0 (0.0)	+
Fa0225022	1.0 (0.0)	1.0 (0.0)	—
Fg0407011	13.0 (0.0)	13.0 (0.0)	+
Mock inoculation	0.5 (0.0)	0.5 (0.0)	

a Pathogenicity was evaluated by the number of florets showing symptoms (bleaching and/or necrosis) at 9 d after inoculation. Three pots were used for a transformant in a trial. Partial bleaching appeared in a floret with mock inoculation, and it was exceptionally evaluated as 0.5.

b Perithecium formation was assayed on a carrot agar plate. Perithecia were formed (+) or not formed (−) by 3 wk after knockdown.

### FGSG_02810 gene disruption in the wild-type strain

Disruption of FGSG_02810 was carried out to confirm that this gene is involved in both pathogenicity and perithecium formation in the *Fg* complex. Three disruption mutants of Fg0407011 were obtained by transformation with the replacement vector pCR402810dis, containing the hygromycin resistance gene (Hyg^R^).

**Figure 5:**
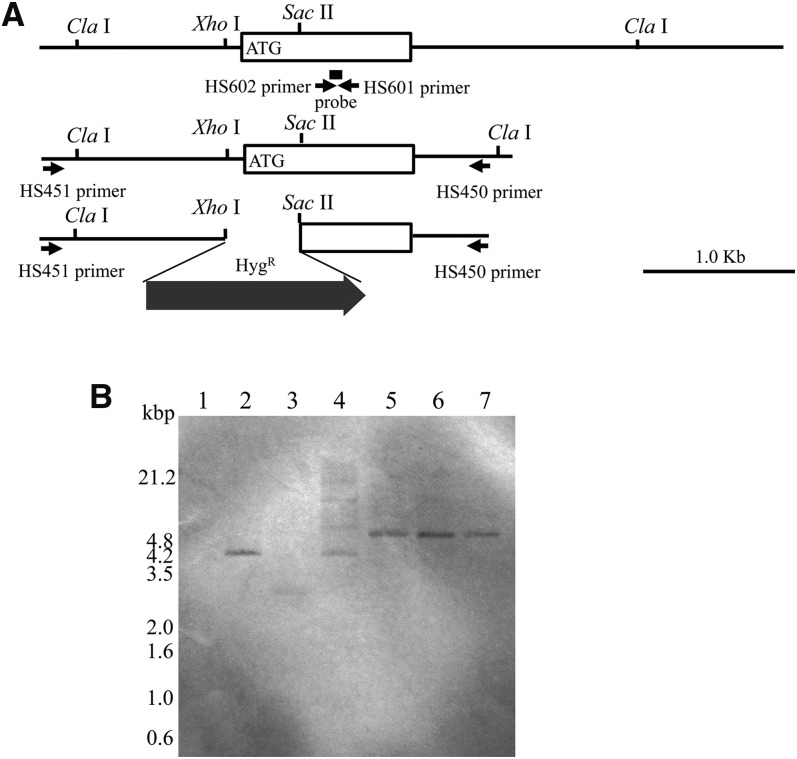
Southern blot analysis. (A) Schematic depiction of the FGSG_02810 gene locus. The open reading frame of FGSG_02810 is indicated by the white box; ATG is the start codon. The restriction site and primer position used for the construction replacement vector pCR402810dis and Southern blot analysis are indicated by horizontal lines and arrows, respectively. The genomic context of Fg0407011 and the probe region between primer HS601 and HS602 for Southern blot analysis are shown at the top. The PCR product of Fg0407011 obtained with the primer pair HS450/HS451 was cloned into pCR4TOPO, and the context of the resulting vector pCR402810 is shown in the middle. The 0.5-kb *Xho*I and *Sac*II fragment in pCR402810 was replaced with a 1.5-kb hygromycin resistance cassette (Hyg^R^), and the context of the resulting vector pCR402810dis is shown at the bottom. (B) Southern blot analysis probed with the PCR product obtained with primer pair HS601/HS602. Genomic DNA was digested with *Cla*I. Shown are the mutant strain Fa0225022 (lane 1); wild-type strain Fg0407011 (lane 2); an FGSG_02810 gene integration transformant of Fa0225022 with pCR402810, Fa02810-1 (lane 3); an ectopic transformant of Fg0407011 with pCR402810dis, Fg02810dis-1 (lane 4); and FGSG_02810 gene disruption transformants of Fg0407011 with pCR402810dis: Fg02810dis-5 (lane 5), Fg02810dis-8 (lane 6), and Fg02810dis-36 (lane 7).

Gene disruption was confirmed by PCR and Southern blot analysis using a probe for part of the FGSG_02810 gene (Figure 5). The disruption mutants showed a 4.7-kb hybridizing band instead of the 3.7-kb band in the wild-type strain (Figure 5). One ectopic transformant (Fg02810dis-1), and one FGSG_02810 gene complementation transformant (Fa02810-1), were also analyzed by Southern blot. The ectopic transformant showed several hybridization signals, in addition to the 3.7-kb band. Fa0225022 did not show any hybridization signal because the probe region is missing in this strain, while the FGSG_02810 gene complementation transformant showed a single hybridization band with *Cla*I digestion (Figure 5). The ectopic transformant showed a phenotype similar to the wild-type strain Fg0407011, namely, pathogenic and perithecium formation. The FGSG_02810 gene disruption mutants showed phenotypes similar to Fa0225022, namely, nonpathogenic and nonperithecium formation (Table 1).

### Expression of the FGSG_02810

**Figure 6:**
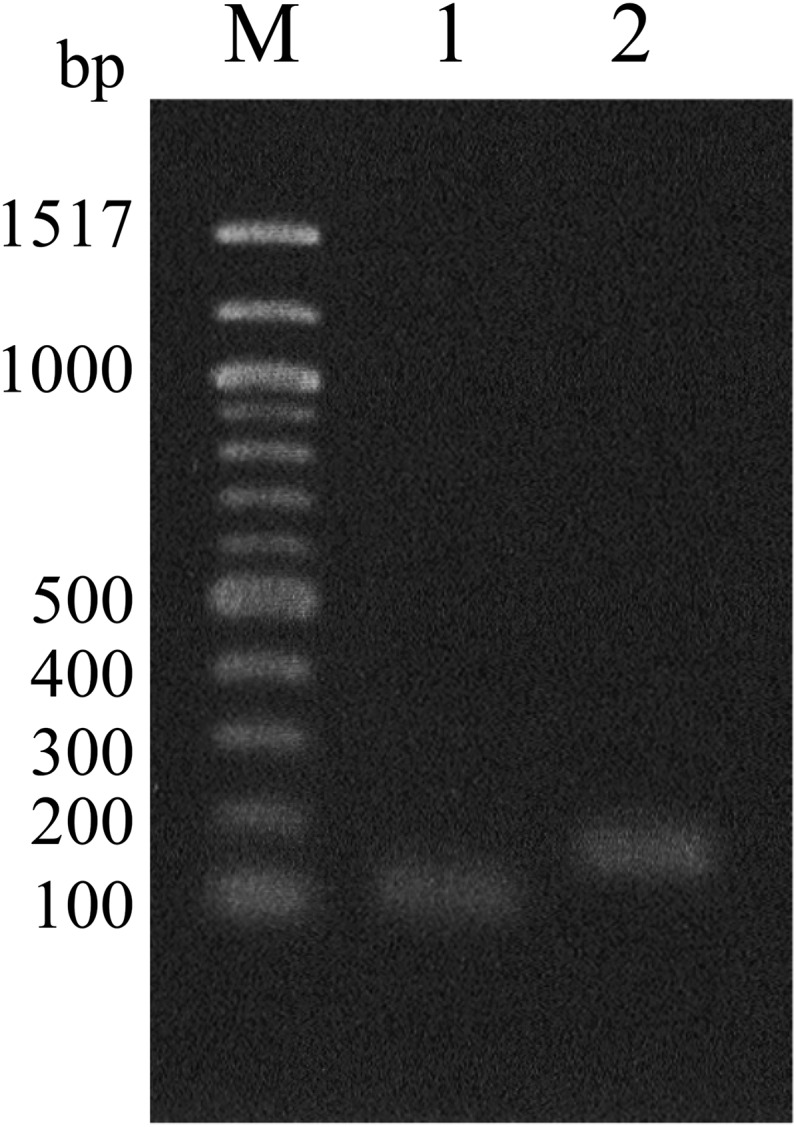
Expression of the FGSG_02810 gene. Transcription of FGSG_02810 in the wild-type strain Fg0407011 was confirmed by RT-PCR with the primer pair HS601/HS602. Shown are size markers (lane M), RT-PCR product obtained with total RNA isolated from Fg0407011 mycelium grown on PDB (lane 1), and RT-PCR product obtained with genomic DNA as a template as control (lane 2).

Transcription of FGSG_02810 in the wild-type strain Fg0407011 was confirmed by RT-PCR using HS601 and HS602 primers. Total RNA was isolated from the wheat spikelet infected with Fg0407011, and also from Fg0407011 mycelium grown on PDB. Both samples showed a 130-bp PCR product, corresponding to the size of the genomic DNA without a 56-bp intron (Figure 6).

### Function prediction of the FGSG_02810

A conserved domain for the medium chain reductase/dehydrogenase (MDR)/zinc-dependent alcohol dehydrogenase-like family was detected at amino acids 134–214 of FGSG_02810 in strain Fg0407011 by using the conserved domain search in the National Center for Biotechnology Information (NCBI) database (2.20 e^−03^) (Figure S1) (Marchler-Bauer *et al.* 2015). The 17 N-terminal amino acids of FGSG_02810 were predicted as a signal peptide with SignalP 4.0 (D-Score: 0.834) (Petersen *et al.* 2011).

BLAST P search in the NCBI database indicated that FGSG_02810 has homology to the gene product of other *Fusarium* spp. for which whole genome sequences have been obtained, including *F. pseudograminearum* FPSE_05581, *F. avenaceum* FAVG1_10471, *F. oxysporum* FOXB_02761, *F. oxysporum* f. sp. *cubense* race 4 FOC4_g10001017, *F. verticillioides* FVEG_13122, *F. fujikuroi* FFUJ_05566, and *Nectria hematococca* mpVI NECHADRAFT_92223 (E value: 2e^−165^ to 2e^−116^), although the function of these proteins has not been determined. FGSG_02810 also has homology to the gene product of other ascomycetous fungi, *Togninia minima* XP_007917001.1 (2e^−107^) and *Beauveria bassiana* XP_008598424.1 (2e^−101^), both of which are putative glycosylphosphatidylinositol (GPI) anchored proteins. A GPI-modification site search with big-PI Fungal Predictor (Eisenhaber *et al.* 2004) indicated that serine 206 and glycine 207 in FGSG_02810 are predicted GPI-modification sites (p-value: 3.1e^−05^) (Figure S1).

## Discussion

A natural mutation involving both pathogenicity and perithecium formation of the *Fg* complex has been revealed in this study. A deletion of ∼3 kb in chromosome 2 was detected in the Fa0225022 mutant, and a lack of the FGSG_02810 gene encoding a putative GPI-anchored protein in the corresponding genomic region was shown to cause the deficiency of pathogenicity and perithecium formation in this strain. As far as we know, this is the first report to clarify a natural mutation responsible for the deficiency of both pathogenicity and perithecium formation in the *Fg* complex.

Previously, artificial random plasmid insertions in the genome, gene disruption, and transposon tagging (Dufresne *et al.* 2008) have been used to identify genes involved *Fg* complex pathogenicity. Pathogenicity of the *Fg* complex has been shown to involve genes for signal transduction (Jenczmionka *et al.* 2003; Hou *et al.* 2002; Yu *et al.* 2008), nonribosomal peptide synthetases (Lu *et al.* 2003), and amino acid synthase (Seong *et al.* 2005; Seo *et al.* 2007).

As summarized by Urban and Hammond-Kosack (2013), the artificial gene disruption method (Hou *et al.* 2002; Jenczmionka *et al.* 2003; Shim *et al.* 2006) has been used to identify 159 genes that contribute to the pathogenicity of the *Fg* complex, as well as several genes involved in both pathogenicity and perithecium formation. However, the contribution of FGSG_02810 to pathogenicity and perithecium formation has not previously been reported.

Cumagun *et al.* (2004) performed linkage mapping of pathogenicity with a natural mutant and observed close linkage between the pathogenicity locus (PATH1) and the perithecium formation locus (PERI1), although the gene(s) was not revealed. In addition to Fa0225022, we found another nonpathogenic mutant strain (Fa0233007) in our *Fg* complex collection (Suga *et al.* 2008), and it is also unable to form the perithecium. However, phenotype recovery by transformation of this mutant strain with pCB02810 failed, and successful PCR amplification of FGSG_02810 in the mutant strain (data not shown), suggested that another gene mutation is responsible for nonpathogenic and nonperithecium formation in Fa0233007.

The FGSG_02810 gene encodes a putative GPI anchor protein. GPI anchor proteins are known to be present in the cell wall (Kapteyn *et al.* 1996; Schoffelmeer *et al.* 2001; De Groot *et al.* 2005) and may be involved in hyphal fusion. Disruption of the mitogen-activated protein (MAP) kinase genes MGV1 and Gpmk1 in the *Fg* complex was found to result in deficiencies of both pathogenicity and perithecium formation (Hou *et al.* 2002; Jenczmionka *et al.* 2003). Modification of cell wall structure and loss of self-hyphal fusion were observed in the MGV1 disruption mutant, and this mutant was both female-sterile and male-fertile (Hou *et al.* 2002). Similarly, Fa0225022 and the natural mutant used in the study of Cumagun *et al.* (2004) were female-sterile and male-fertile because the outcrossing succeeded. Some *Fusarium* species are known to have a female sterile strain that is frequently isolated in nature, but the responsible mutation(s) has not yet been revealed. Mutation of a FGSG_02810 homolog is a possible candidate for female sterilization, although natural female sterile strains are uncommon in the *Fg* complex.

The involvement of a GPI anchor protein gene in pathogenicity has been revealed in the rice fungal pathogen *Pyricularia oryzae* (Ahn *et al.* 2004), and the human fungal pathogen *Aspergillus fumigatus* (Li *et al.* 2007). GPI anchor protein genes, FGSG_00576, FGSG_001588, and FGSG_08844, are predicted in *F. graminearum* s. str., but their disruption did not alter the phenotype (including pathogenicity), although disruption of the phosphoethanoleamine transferase gene (*gpi7*) resulted in cell wall structure abnormalities, macroconidium production, and pathogenicity to wheat in *F. graminearum* s. str. (Rittenour and Harris 2013).

Rittenour and Harris (2013) detected 57 genes encoding putative GPI anchor proteins in the *F. graminearum* s. str. genome that are similar to proteins with known functions; several genes encode putative carbohydrate-modifying enzymes. A conserved domain for the medium chain reductase/dehydrogenase (MDR)/zinc-dependent alcohol dehydrogenase-like family was also detected in FGSG_02810, although this gene was not included by Rittenour and Harris (2013). According to Rittenour and Harris (2013), no phenotypic change could be due to the gene redundancy of FGSG_001588 and FGSG_08844, while our results indicate that FGSG_02810 is a single gene. The *gpi7* might affect FGSG_02810 gene function, although alteration of macroconidium production was not detected in the FGSG_02810 complement transformant or FGSG_02810 disruption mutants (data not shown).

Natural pathogenic variation has been observed in the *Fg* complex, but the cause of this variation is poorly understood at a molecular level. In this study, using genetic mapping aided by whole genome sequence (Cuomo *et al.* 2007), we identified a natural mutation involving pathogenicity of the *Fg* complex. Although the function of FGSG_02810 remains unclear, our data suggest that it plays a role in breaking the rachis nodes in wheat head and perithecium formation.

Further study will lead to a better understanding of the molecular mechanisms involved in host infection and production of inoculum in the *Fg* complex. Considerable bioinformatics analysis data (such as transcriptome and proteome) are now available on the *Fg* complex. Moreover, combinational analyses of genetic mapping and bioinformatics data are also useful for further elucidation of phenotypic variations of the *Fg* complex at the molecular level.

## Supplementary Material

Supplemental Material
