# Supplementary material for: A Natural Mutation Involving both Pathogenicity and Perithecium Formation in the Fusarium graminearum Species Complex
Source: G3 (Bethesda). 2016 Sep 27;6(12):3883–92. doi: 10.1534/g3.116.033951 (PMC5144959; doi:10.1534/g3.116.033951)
Supplement: Supplemental Material [file supp_6_12_3883__index.html]

A Natural Mutation Involving both Pathogenicity and Perithecium Formation in the Fusarium graminearum Species Complex — Supplemental Material 

# A Natural Mutation Involving both Pathogenicity and Perithecium Formation in the *Fusarium graminearum* Species Complex

## Supplemental Material for Suga *et al.*, 2016

**Files in this Data Supplement:**

- Figure S1 - The FGSG\_02810 gene (.ppt, 131 KB)
- Table S1 - Primers used in this study. (.xls, 56 KB)
