# Supplementary material for: A Natural Mutation Involving both Pathogenicity and Perithecium Formation in the Fusarium graminearum Species Complex
Source: G3 (Bethesda). 2016 Sep 27;6(12):3883–92. doi: 10.1534/g3.116.033951 (PMC5144959; doi:10.1534/g3.116.033951)
Supplement: Supplemental Material [file supp_g3.116.033951_FigureS1.ppt]

## Slide 1
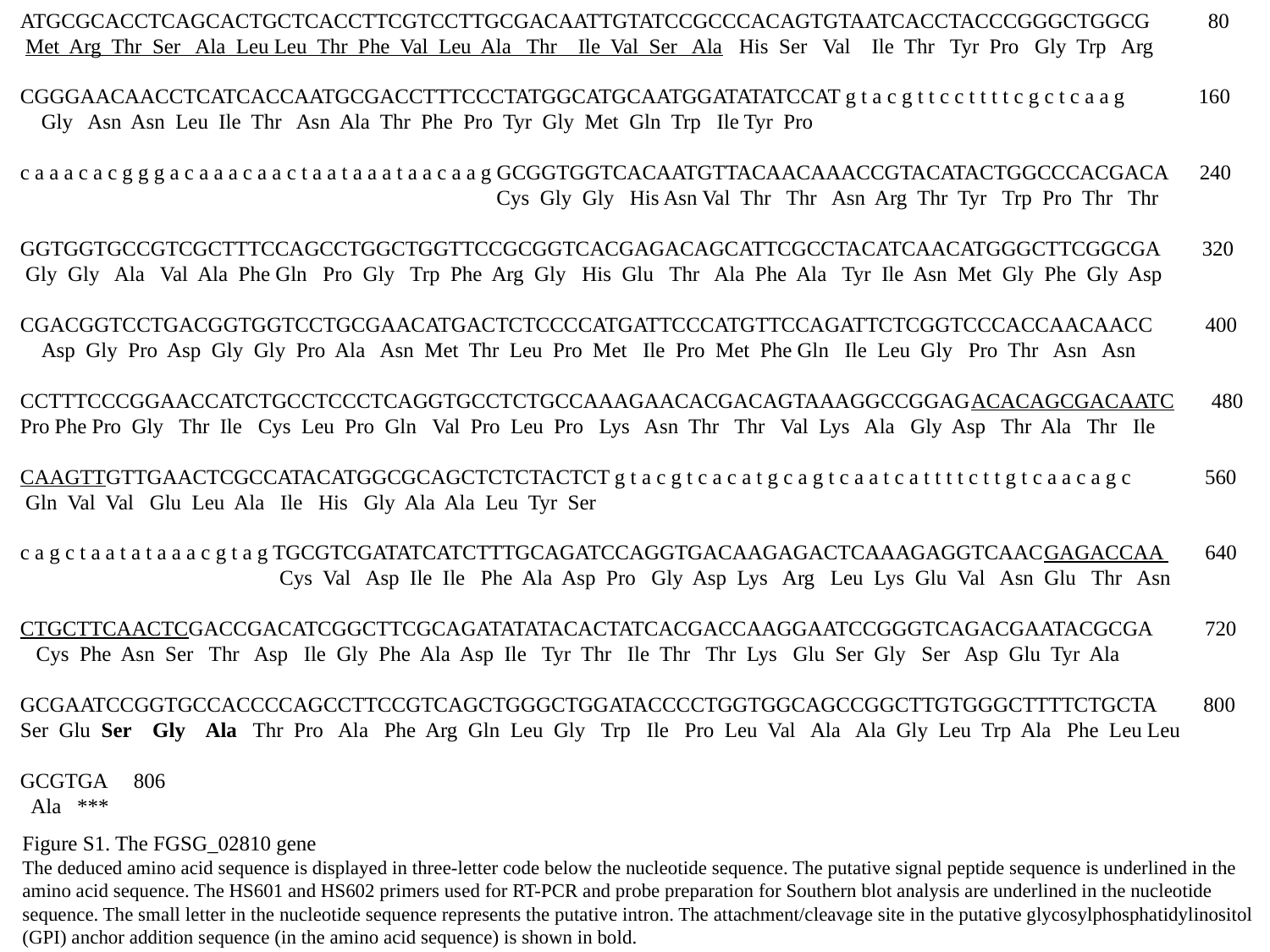

ATGCGCACCTCAGCACTGCTCACCTTCGTCCTTGCGACAATTGTATCCGCCCACAGTGTAATCACCTACCCGGGCTGGCG 80
 Met Arg Thr Ser Ala Leu Leu Thr Phe Val Leu Ala Thr Ile Val Ser Ala His Ser Val Ile Thr Tyr Pro Gly Trp Arg
CGGGAACAACCTCATCACCAATGCGACCTTTCCCTATGGCATGCAATGGATATATCCAT g t a c g t t c c t t t t c g c t c a a g 160
 Gly Asn Asn Leu Ile Thr Asn Ala Thr Phe Pro Tyr Gly Met Gln Trp Ile Tyr Pro
c a a a c a c g g g a c a a a c a a c t a a t a a a t a a c a a g GCGGTGGTCACAATGTTACAACAAACCGTACATACTGGCCCACGACA 240
 Cys Gly Gly His Asn Val Thr Thr Asn Arg Thr Tyr Trp Pro Thr Thr
GGTGGTGCCGTCGCTTTCCAGCCTGGCTGGTTCCGCGGTCACGAGACAGCATTCGCCTACATCAACATGGGCTTCGGCGA 320
 Gly Gly Ala Val Ala Phe Gln Pro Gly Trp Phe Arg Gly His Glu Thr Ala Phe Ala Tyr Ile Asn Met Gly Phe Gly Asp
CGACGGTCCTGACGGTGGTCCTGCGAACATGACTCTCCCCATGATTCCCATGTTCCAGATTCTCGGTCCCACCAACAACC 400
 Asp Gly Pro Asp Gly Gly Pro Ala Asn Met Thr Leu Pro Met Ile Pro Met Phe Gln Ile Leu Gly Pro Thr Asn Asn
CCTTTCCCGGAACCATCTGCCTCCCTCAGGTGCCTCTGCCAAAGAACACGACAGTAAAGGCCGGAGACACAGCGACAATC 480
Pro Phe Pro Gly Thr Ile Cys Leu Pro Gln Val Pro Leu Pro Lys Asn Thr Thr Val Lys Ala Gly Asp Thr Ala Thr Ile
CAAGTTGTTGAACTCGCCATACATGGCGCAGCTCTCTACTCT g t a c g t c a c a t g c a g t c a a t c a t t t t c t t g t c a a c a g c 560
 Gln Val Val Glu Leu Ala Ile His Gly Ala Ala Leu Tyr Ser
c a g c t a a t a t a a a c g t a g TGCGTCGATATCATCTTTGCAGATCCAGGTGACAAGAGACTCAAAGAGGTCAACGAGACCAA 640
 Cys Val Asp Ile Ile Phe Ala Asp Pro Gly Asp Lys Arg Leu Lys Glu Val Asn Glu Thr Asn
CTGCTTCAACTCGACCGACATCGGCTTCGCAGATATATACACTATCACGACCAAGGAATCCGGGTCAGACGAATACGCGA 720
 Cys Phe Asn Ser Thr Asp Ile Gly Phe Ala Asp Ile Tyr Thr Ile Thr Thr Lys Glu Ser Gly Ser Asp Glu Tyr Ala
GCGAATCCGGTGCCACCCCAGCCTTCCGTCAGCTGGGCTGGATACCCCTGGTGGCAGCCGGCTTGTGGGCTTTTCTGCTA 800
Ser Glu Ser Gly Ala Thr Pro Ala Phe Arg Gln Leu Gly Trp Ile Pro Leu Val Ala Ala Gly Leu Trp Ala Phe Leu Leu
GCGTGA 806
 Ala ***
Figure S1. The FGSG_02810 gene
The deduced amino acid sequence is displayed in three-letter code below the nucleotide sequence. The putative signal peptide sequence is underlined in the amino acid sequence. The HS601 and HS602 primers used for RT-PCR and probe preparation for Southern blot analysis are underlined in the nucleotide sequence. The small letter in the nucleotide sequence represents the putative intron. The attachment/cleavage site in the putative glycosylphosphatidylinositol (GPI) anchor addition sequence (in the amino acid sequence) is shown in bold.
